# Optical coherence tomography angiography vessel density in children with type 1 diabetes

**DOI:** 10.1371/journal.pone.0186479

**Published:** 2017-10-20

**Authors:** Joanna Gołębiewska, Andrzej Olechowski, Marta Wysocka-Mincewicz, Dominik Odrobina, Marta Baszyńska-Wilk, Artur Groszek, Mieczysław Szalecki, Wojciech Hautz

**Affiliations:** 1 Department of Ophthalmology, The Children's Memorial Health Institute, Aleja Dzieci Polskich 20, Warsaw, Poland; 2 Department of Diabetology and Endocrinology The Children's Memorial Health Institute, Aleja Dzieci Polskich 20, Warsaw, Poland; 3 Department of Medicine and Health Sciences, Jan Kochanowski Memorial University of Kielce, Kielce, Poland; 4 Ophthalmology Clinic of St. John Boni Fratres Lodziensis, Łódź, Poland; Weill Cornell Medical College, QATAR

## Abstract

**Purpose:**

To assess the optical coherence tomography angiography (OCTA) retinal vessel density and foveal avascular zone (FAZ) in children with type 1 diabetes (T1D) and compare potential pathologic early changes in this population to healthy age-matched controls.

**Methods:**

This study included 130 pubescent children: 94 with T1D (188 eyes) and 36 of their age-matched control group (60 eyes). OCTA was performed using AngioVue (Avanti, Optivue). FAZ area (mm^2^) in superficial plexus, whole superficial capillary vessel density (wsVD), fovea superficial vessel density (fsVD), parafovea superficial vessel density (psVD), whole deep vessel density (wdVD), fovea deep vessel density (fdVD), parafovea deep vessel density (pdVD), foveal thickness (FT) (μm) and parafoveal thickness (PFT) (μm) were taken into analysis. Among the studied patients with T1D there were assessed codependences regarding the investigated foveal and parafoveal parameters and selected potential predictors, i.e. patient’s age (years), diabetes duration time (years), age of onset of the disease (years), mean level of glycated hemoglobin (HbA1C) (%), and concentration of serum creatinine (mg/dL).

**Results:**

None of the abovementioned OCT and OCTA parameters was statistically significantly different between the groups. The patient’s age statistically significantly did not influent any of the OCT and OCTA parameters. Yet an elevated level of HbA1C tended to reduce the parafovea superficial vessel density (p = 0.039), and parafoveal thickness (p = 0.003) and an increased serum creatinine level correlated with the decreased whole deep vessel density (p < 0.001). The parafovea deep vessel density in the diabetic patients decreased when the serum creatinine level (p = 0.008), age of onset of the disease (p = 0.028), and diabetes duration time (p = 0.014) rose.

**Conclusions:**

Vessel density, both in superficial and deep plexuses, and FAZ area are normal in pubescent children with T1D comparing to healthy subjects. An elevated level of HbA1C correlated with reduced psVD and PFT. Longitudinal observation of these young patients is needed to determine if any of these OCTA measurements are predictive of future DR severity.

## Introduction

Diabetes mellitus is the third most common chronic disease among children. Pediatric populations would appear to be at low risk for DR, but some adolescents develop either clinically significant macular edema or even proliferative retinopathy. [1–4] Pubertal status and the prepubertal duration of diabetes influence the risk of developing DR, as children under the age of 10 years have minimal risk, and no cases of proliferative DR in the first decade of life were noted. [5–7] Therefore, early detection of DR through screening programs is crucial for preserving vision in patients with diabetes. [8,9]

Indirect ophthalmoscopy and stereoscopic fundus photography through dilated pupils, a practice commonly used worldwide, provides the great diagnostic accuracy in detecting DR. [9,10] Over the years fluorescein angiography (FA) is the gold standard in diabetic retinopathy diagnosis and classification, but this method requires an intravenous dye injection and causes significant discomfort and stress. [11,12] Optical coherence tomography angiography (OCTA) is a new, non-invasive tool, based on split-spectrum amplitude-decorrelation angiography (SSADA), involving the detection and measurement of intravascular erythrocyte movement. [13] OCTA enables reproducible, quantitative assessment of the microcirculation in the macula and in the optic nerve head and may be used in diagnosing of glaucoma and different retinal vascular diseases, such as diabetic retinopathy, retinal vein occlusion, central serous chorioretinopathy and age-related macular degeneration. [14–16] OCTA provides three-dimensional maps of the macular perfusion and seems to be promising method in the detection of early microcirculation disorders. To date, there are a lot of studies focus on OCTA in adults with diabetes mellitus. [17–20] To the best of our knowledge there are only few studies on OCTA in the diagnostics of pediatric patients and there have been no previous reports on OCTA findings in pediatric population with T1D. [21,22]

The aim of the present study was to assess the OCTA retinal vessel density and FAZ area in children with T1D and compare potential pathologic early changes in this population to healthy age matched controls.

## Methods

This prospective, observational study was conducted in The Children's Memorial Health Institute in Warsaw and enrolled all consecutive patients available between March 2015 and September 2016 in the Department of Endocrinology and Diabetology, who met inclusion criteria. The study was approved by the Bioethics Committee The Children's Memorial Health Institute in Warsaw and followed the tenets of the Declaration of Helsinki. A written informed consent was obtained from the patient’s legal guardian and from patients > 16 years old after explanation of the nature of the non-invasive study. Inclusion criteria were age ≥11 years and diabetes duration ≥ 1 year. Exclusion criteria were history of prematurity, other concomitant retinal pathologies, such as hereditary retinal dystrophies, vitreoretinal diseases, pathologic myopia (defined as a spherical equivalent of −6 diopters or more), history of uveitis. Eyes with poor quality scans were also excluded. Clinicopathological data recorded for each diabetic subject included duration of diabetes, systolic and diastolic blood preasure, glycated hemoglobin (HbA1C) levels, creatinine in the daily collection of urine and serum creatinine levels, microalbuminuria level. Control subjects were defined as having a normal ophthalmic examination and no history of diabetes. Every patient underwent a complete ophthalmological examination, including best-corrected visual acquity (BVCA), slit-lamp biomicroscopy, dilated fundus examination and color fundus photography.

OCTA was performed using a commercially available RTVue XR Avanti with AngioVue (Optovue, Fremont, CA, USA) with 3 mm x3 mm images of the macula, centered on the foveola. Each OCTA en face image contains 304 x 304 pixels created from the intersection of the 304 vertical and the 304 horizontal B-scans. AngioVue automatically segments the area into four layers, including superficial capillary plexus layer (SP), deep capillary plexus layer (DP), outer retina layer and choriocapillaries. The SP en face image was segmented with an inner boundary at 3 μm beneath the internal limiting membrane and an outer boundary set at 15 μm beneath the inner plexiform layer, whereas the deep capillary plexus en face image was segmented with an inner boundary 15 μm beneath the inner plexiform layer and an outer boundary at 70 μm beneath the inner plexiform layer. Integrated automated algorithms provided by the machines software were used to quantify FAZ area (mm^2^) and macular vascular density (%). FAZ area was automatically calculated for superficial plexus. Capillary vascular density in macular and paramacular region were measured both in superficial and deep plexuses. Vessel density is calculated as the percentage area occupied by flowing blood vessels in the selecting region, which enable of quantitative assessment of microvasculature. Whole superficial capillary vessel density (wsVD), fovea superficial vessel density (fsVD), parafovea superficial vessel density (psVD), whole deep vessel density (wdVD), fovea deep vessel density (fdVD), parafovea deep vessel density (pdVD) were taken into analysis. Foveal thickness (FT) (μm) and parafoveal thickness (PFT) (μm) data were obtained from retinal maps, using the same device. All subjects were dilated with 1% tropicamide eye drops before examination. Stabilization was achieved with the standard chinrest and forehead support. Subjects were directed to focus on an internal fixation target. Three scans for each eye were captured, then the best one in quality (with a signal strength index >60) was considered for analysis. Two trained OCTA readers (JG, AO) reviewed all images independently to ensure correct segmentation and identify poor quality scans, with motion artifacts or blurred images, where data were insufficient for proper analysis. The data collected from both eyes of the studied patients were taken into analysis.

### Statistical analysis

The investigated traits were described by way of measures of location–mean, median and quartiles, along with measures of dispersion–interquartile range, standard deviation, standard error of mean, 95% confidence interval, and minimum-to-maximum values.

Differences in the investigated parameters between study groups were tested by using the multifactor analysis of variance (ANOVA) without replication or generalized linear models with intra-subject standard errors.

A level of P < 0.05 was considered statistically significant. All the statistical computations were carried out by means of Stata/Special Edition, release 14.2 (StataCorp LP, College Station, Texas, USA).

## Results

A 212 eyes of 106 pediatric patients with T1D were recruited to this study. After exclusion of eyes with poor quality OCTA images (12 patients, 24 eyes), 94 diabetic children (188 eyes) and 36 (60 eyes) their age-matched control group were taken to final analysis. The mean age of the diseased participants amounted to 15.3 (± SD = 2.1) years and in the control group–to 13.6 (± SD = 1.8) years (P < 0.001). The 94 study participants had been diagnosed with T1D, on average, 6.4 (± SD = 3.3) years earlier. The color fundus photographs were normal in all subjects and BCVA was 20/20.

The characteristics of the all participants is summarized in Table 1.

**Table 1:** Characteristics of the all participants.

Investigated trait	Statistical parameter						
	M*	*Me*^*†*^	*Q*_*1*_ *–Q*_*3*_^*‡*^ (IQR**)	*SD*^*††*^	*SE*^*‡‡*^	95% CI***	*Min*.*–max*.
Age (years) - DM (+) - DM (-)	15.3 13.6	15.7 13.0	13.4–17.3 (3.9) 12.0–15.0 (3.0)	2.1 1.8	0.2 0.3	14.9–15.7 13.1–14.2	11.3–18.5 11.0–18.0
Diabetes duration time (years)	6.4	6.2	4.0–8.6 (4.6)	3.3	0.3	5.8–7.1	1.0–14.4
Age of onset DM (years)	8.9	9.2	6.0–11.4 (5.4)	3.8	0.3	8.2–9.6	2.3–16.5
Glycated hemoglobin –actual level (%)	8.2	8.0	7.4–8.8 (1.4)	1.3	0.1	7.9–8.5	6.4–13.3
Glycated hemoglobin –mean level (%)	8.1	8.0	7.2–8.5 (1.3)	1.1	0.1	7.8–8.3	6.3–11.6
Microalbuminuria –actual level (mg/d)	10.8	9.3	6.7–12.9 (6.2)	7.2	0.8	9.2–12.3	0.5–33.3
Microalbuminuria –mean level (mg/d)	9.2	6.5	4.0–11.5 (7.5)	8.4	0.9	7.3–11.0	0.5–40.5
Serum creatinine (mg/dL)	0.69	0.67	0.59–0.78 (0.19)	0.15	0.02	0.66–0.72	0.42–1.12
Creatinine in the daily collection of urine (mg/d)	1.24	1.02	0.83–1.47 (0.64)	0.84	0.10	1.03–1.45	0.27–5.47
Systolic blood pressure (mmHg)	113.4	113.0	105.0–123.0 (18.0)	11.2	1.3	110.8–115.9	84.0–140.0
Diastolic blood pressure (mmHg)	68.4	69.0	60.0–75.0 (15.0)	10.3	1.2	66.1–70.7	40.0–91.0

(* M–mean; ^†^ Me–median; ^‡^ Q–quartiles; ** IQR–interquartile range; ^††^ SD–standard deviation; ^‡‡^ SE–standard error; *** CI–confidence interval)

Descriptive measures for investigated ophthalmic parameters in both study groups are summarized in Table 2.

**Table 2:** Descriptive statistics for investigated ophthalmic parameters in the studied patients by presence of type 1 diabetes mellitus.

Investigated trait	Study group	Statistical parameter							Level of statistical significance
		*M*	*Me*	*Q*_*1*_ *–Q*_*3*_ *(IQR)*	*SD*	*SE*	*95% CI*	*Min*.*–max*.	
Foveal avascular zone, FAZ (mm^2^)	DM (+)	0.231	0.218	0.167–0.300 (0.133)	0.100	0.010	0.211–0.252	0.033–0.550	P = 0.089
	DM (-)	0.240	0.253	0.198–0.284 (0.086)	0.078	0.012	0.216–0.264	0.007–0.386	
Whole superficial vessel density, wsVD (%)	DM (+)	51.98	51.98	50.61–53.60 (2.99)	2.43	0.25	51.48–52.48	45.30–57.38	P = 0.741
	DM (-)	52.45	52.92	50.86–53.85 (2.99)	2.74	0.42	51.60–53.31	46.60–60.25	
Fovea superficial vessel density, fsVD (%)	DM (+)	32.51	31.50	28.78–36.14 (7.36)	5.26	0.54	31.43–33.59	20.77–44.59	P = 0.312
	DM (-)	32.48	32.38	28.69–35.63 (6.94)	5.33	0.82	30.82–34.14	22.90–48.57	
Parafovea superficial vessel density, psVD (%)	DM (+)	53.80	53.91	52.12–55.34 (3.22)	2.54	0.26	53.28–54.32	46.28–59.80	P = 0.986
	DM (-)	54.41	54.91	52.63–55.96 (3.33)	2.62	0.40	53.60–55.23	48.40–62.08	
Foveal thickness, FT (μm)	DM (+)	254.5	253.0	241.0–267.0 (26.0)	20.0	2.1	250.4–258.6	196–301	P = 0.825
	DM (-)	249.9	250.5	238.0–261.0 (23.0)	19.5	3.0	243.9–256.0	212–299	
Parafoveal thickness, PFT (μm)	DM (+)	317.5	317.0	310.0–326.0 (16.0)	15.9	1.6	314.3–320.8	274–361	P = 0.951
	DM (-)	318.1	318.0	307.0–332.0 (25.0)	17.8	2.7	312.5–323.6	282–354	
Whole deep vessel density, wdVD (%)	DM (+)	58.57	58.68	57.27–59.89 (2.62)	1.94	0.20	58.17–58.96	54.14–63.97	P = 0.273
	DM (-)	58.57	59.75	58.24–60.93 (2.69)	5.03	0.78	57.00–60.13	30.30–63.45	
Fovea deep vessel density, fdVD (%)	DM (+)	32.37	32.15	27.65–36.24 (8.59)	6.17	0.64	31.11–33.63	16.63–45.85	P = 0.169
	DM (-)	31.75	31.92	30.08–33.00 (2.92)	3.96	0.61	30.51–32.99	23.71–48.44	
Parafovea deep vessel density, pdVD (%)	DM (+)	61.28	61.69	59.64–62.46 (2.82)	2.10	0.22	60.85–61.71	54.86–67.21	P = 0.863

(* M–mean; ^†^ Me–median; ^‡^ Q–quartiles; ** IQR–interquartile range; ^††^ SD–standard deviation; ^‡‡^ SE–standard error; *** CI–confidence interval)

**Fig 1:**
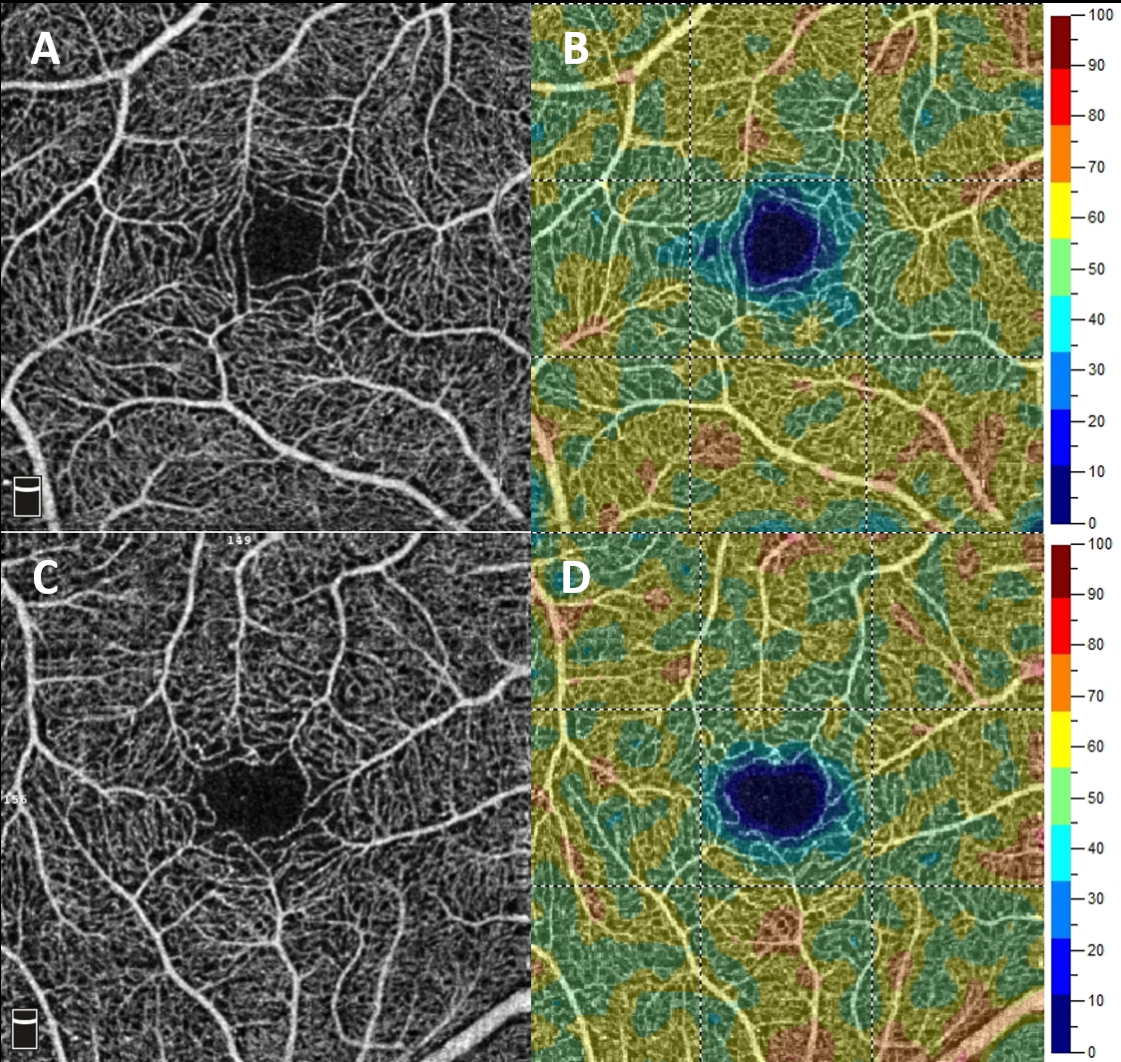
Representative OCTA images of a control subject and a patient with diabetes. A- superficial retinal plexus (signal strength = 82), B- superficial vessel density map of healthy subject (whole vessel density = 57,59%). C- superficial retinal plexus (signal strength = 76), D- superficial vessel density map of diabetic subject (whole vessel density = 56,40%).

**Fig 2:**
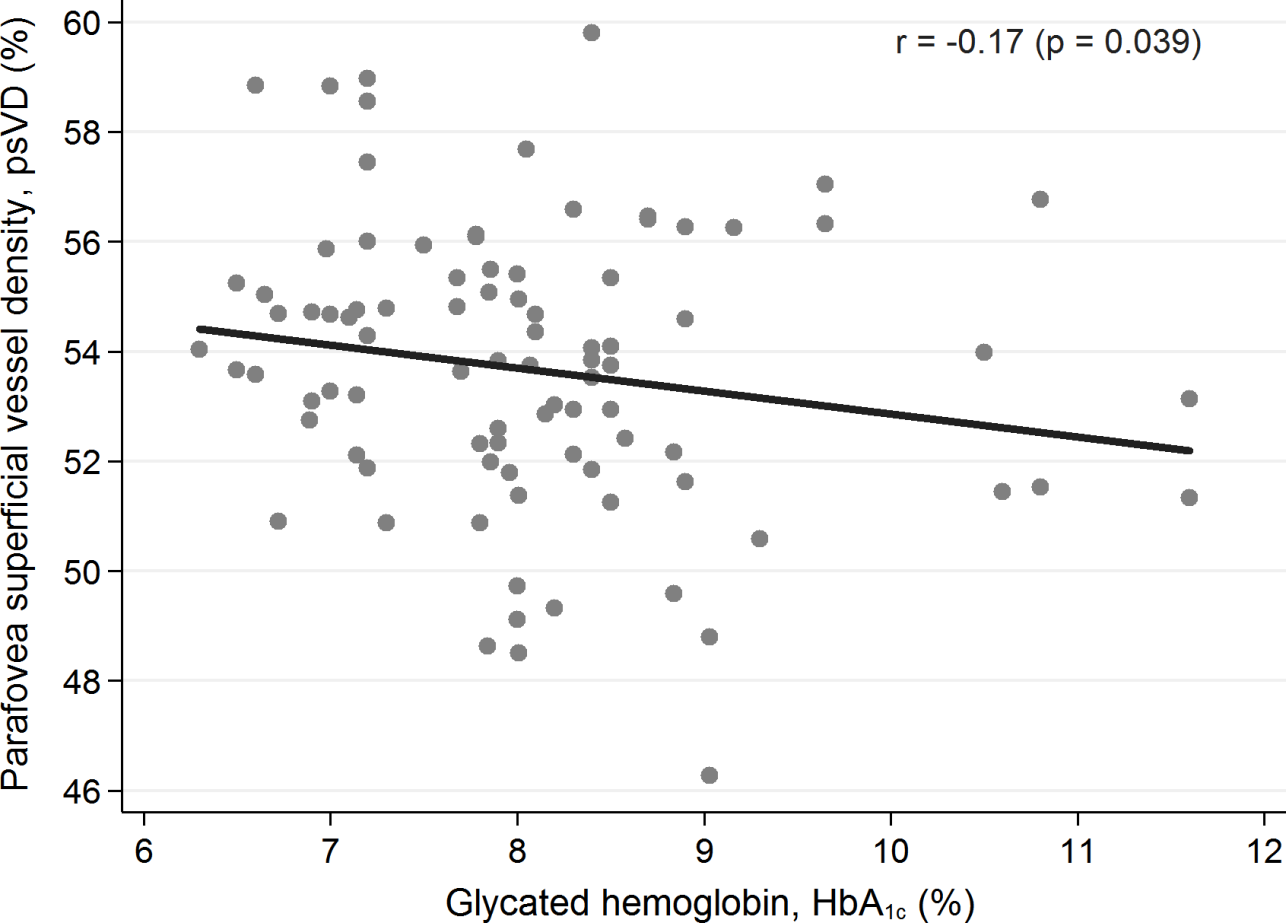
Correlation between parafovea vessel density and HbA1C level among the patients with T1D (p = 0.039).

**Fig 3:**
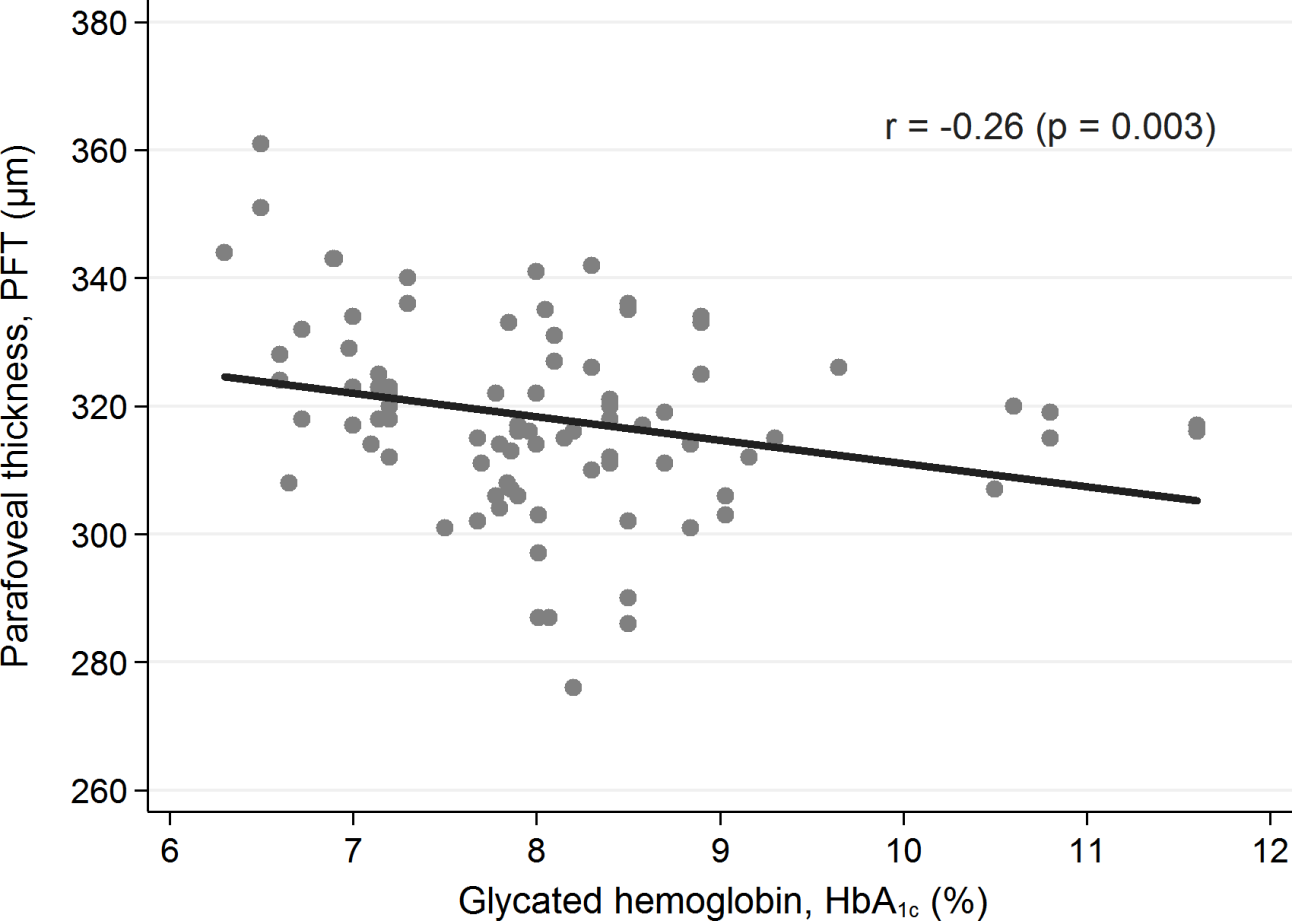
Correlation between parafoveal thickness and HbA1C level in patients with T1D (p = 0.03).

**Fig 4:**
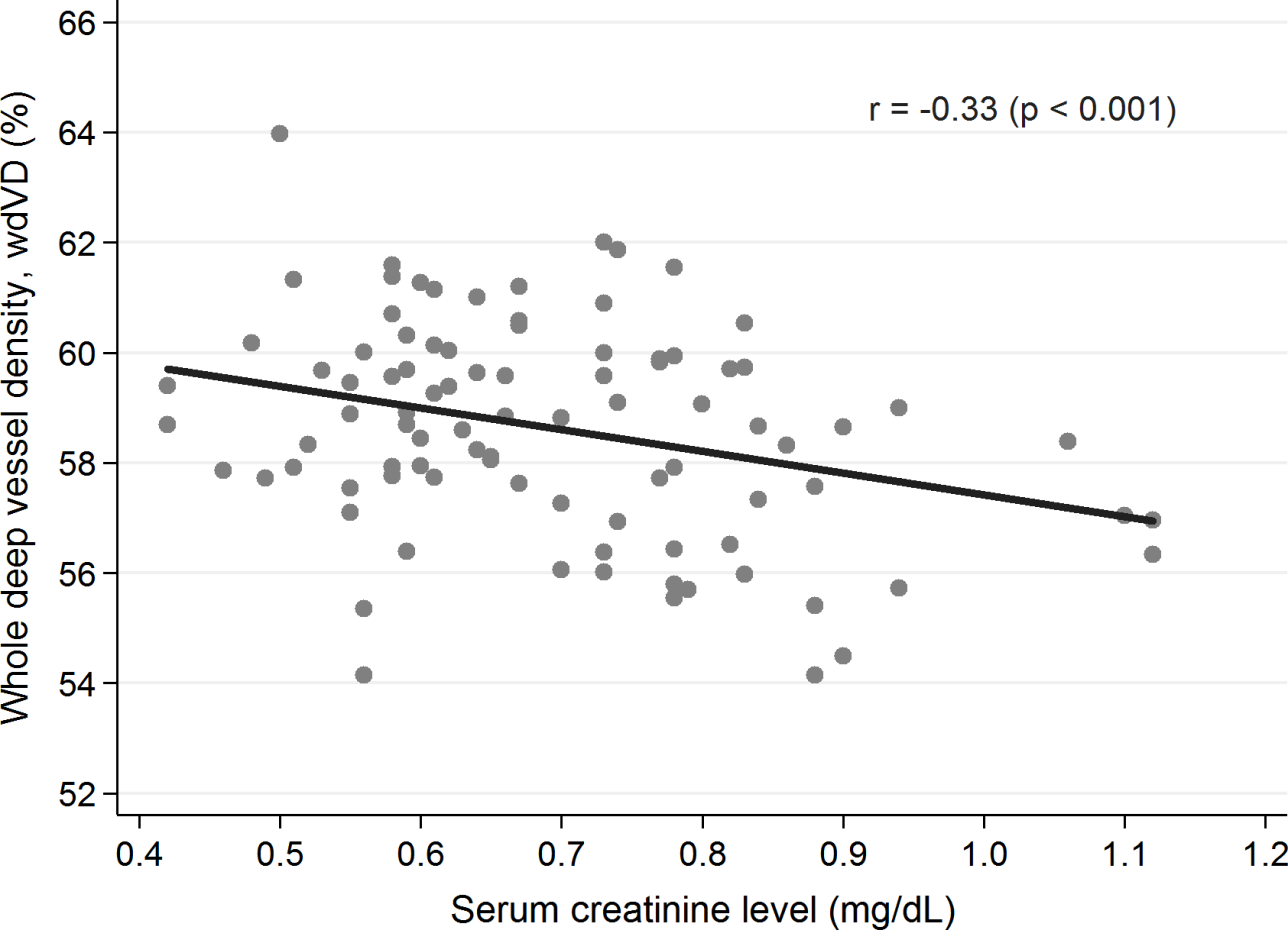
Correlation between whole vessel density (wVD, %) and creatinine serum level in patients with T1D (p<0,001).

None of the abovementioned parameters was statistically significantly different between the groups. Representative OCTA images of control and diabetic subjects demonstrates Fig 1. Among the studied patients with T1D there were assessed codependences regarding the investigated foveal and parafoveal parameters (displayed in Table 2) and selected potential predictors, i.e. patient’s age (years), diabetes duration time (years), age of onset of the disease (years), mean level of glycated hemoglobin (%), and concentration of serum creatinine (mg/dL). The patient’s age statistically significantly did not influent any of the OCT and OCTA parameters. Yet an elevated level of HbA1C tended to reduce the parafovea superficial vessel density (p = 0.039) (Fig 2), and parafoveal thickness (p = 0.003) (Fig 3) and an increased serum creatinine level correlated with the decreased whole deep vessel density (p < 0.001) (Fig 4). The parafovea deep vessel density in the diabetic patients decreased when the serum creatinine level (p = 0.008), age of onset of the disease (p = 0.028), and diabetes duration time (p = 0.014) rose.

## Discussion

Several studies over the past 20–30 years show a declining incidence of DR in children with diabetes, from 49% in the early 1990s to 24% in the early 2000s.

This result may be due to more effective treatment, the use of the insulin pumps and better education of the affected children and their families. [23,24] In our study group no child had any signs of diabetic retinopathy on fundus examination and color fundus photography. As with adults, the risk of developing retinopathy in youths is time dependent, but it appears to be non-linear before puberty, as this period contributes less to the development of DR. [25] Classification of Diabetic Retinopathy from Fluorescein Angiograms Classification of Diabetic Retinopathy from Fluorescein AngiogramsIn this study we performed quantitative analyses of macular vasculature in a group of pubescent subjects with T1D using novel, non-invasive OCTA, which seemed to be an easily accepted method by youths.

Previous studies based on OCTA reported several retinal microvasculature abnormalities in adult diabetic patients, including capillary dropout, tortuous capillary branches, dilated capillary loops, reduced capillary perfusion, microaneurysms, irregular FAZ contour and FAZ enlargement. Severity of these pathologies correlates with severity of the DR. [17–20,26] Choi et al reported capillaries and choriocapillaries alteration not only in patients with proliferative DR (PDR), non-proliferative DR (NPDR) but also in subjects without clinical DR. [27] Our study did not confirm these results in pubescent youths. Several studies using FA and OCTA found enlarged FAZ area as an indicator of early ischemia and predictor of DR progression. [28–31] Arend et al reported the FAZ enlargement in patients with diabetes who exhibited reduced visual acuity. [29] In this study all of the examined eyes had good visual acuity, so we did not analyze the correlation. Our results are consistent with Tam et al, who did not find any significant difference for the FAZ diameters between the control and diabetic eyes using adaptive optics scanning laser. [32]

OCTA offers a quantitative, objective assessment of macular vessel density. Several authors reported decreased vessel density in the superficial and deep vascular plexuses in adults with diabetes, regardless of the presence of diabetic retinopathy, as compared to healthy subjects. Vessels density measurements were lower in more severe disease. [32–35] We found no statistically significant correlation between superficial and deep vessel density between diabetic and healthy children. A multifactorial analysis revealed some significantly negative correlations: duration of diabetes, age of onset of the disease and increased serum creatinine level were associated with decreased parafovea deep vessel density. An elevated level of HbA1C was associated with reduced parafovea superficial vessel density and parafoveal thickness in the diabetic subjects. This result is inconsistent with report of Durbin et al., who did not find any correlations between HbA1C and OCTA parameters in diabetic adults.

Concerning the results, this study revealed much less objective features of microvasculature impairment in youths with T1D than other authors reported in diabetic adults. This might confirm better autoregulation and better state of retinal circulation in children with T1D than in adults, although puberty significantly increases the risk of complications of DM. As some previous studies showed that early retinal capillary closure might be transient and reversible the strict control seems to be crucial to prevent the loss of vision in youths with T1D. [36] The main limitation of the study is poor representativeness of the sample–all subjects were Caucasian, majority of them came from the central part of Poland, and the lack of differences in this clinical population may not reflect the entire cohort of T1D children across the world.

## Conclusions

Vessel density, both in superficial and deep plexuses, and FAZ area are normal in pubescent children with T1D comparing to healthy subjects. An elevated level of HbA1C correlated with reduced psVD and PFT. Longitudinal observation of these young patients is needed to determine if any of these OCTA measurements are predictive of future DR severity.

## Supporting information

S1 File Database. (XLSX)
